# Dietary Inflammatory Index and Incidence of Cardiovascular Disease in the SUN Cohort

**DOI:** 10.1371/journal.pone.0135221

**Published:** 2015-09-04

**Authors:** Raúl Ramallal, Estefanía Toledo, Miguel A. Martínez-González, Aitor Hernández-Hernández, Ana García-Arellano, Nitin Shivappa, James R. Hébert, Miguel Ruiz-Canela

**Affiliations:** 1 Department of Cardiology, Complejo Hospitalario de Navarra, Servicio Navarro de Salud, Pamplona, Spain; 2 IdiSNA, Navarra Institute for Health Research, Pamplona, Spain; 3 Department of Preventive Medicine and Public Health, University of Navarra, Pamplona, Spain; 4 Ciber de Fisiopatología de la Obesidad y Nutrición (CIBERObn), Instituto de Salud Carlos III, Madrid, Spain; 5 Cancer Prevention and Control Program, University of South Carolina, Columbia, SC, United States of America; 6 Department of Epidemiology and Biostatistics, Arnold School of Public Health, University of South Carolina, Columbia, SC, United States of America; GDC, GERMANY

## Abstract

**Background:**

Diet is known to play a key role in atherogenesis and in the development of cardiovascular events. Dietary factors may mediate these processes acting as potential modulators of inflammation. Potential Links between inflammatory properties of diet and the occurrence of cardiovascular events have not been tested previously.

**Objective:**

We aimed to assess the association between the dietary inflammatory index (DII), a method to assess the inflammatory potential of the diet, and incident cardiovascular disease.

**Methods:**

In the prospective, dynamic SUN cohort, 18,794 middle-aged, Spanish university graduates were followed up for 8.9 years (median). A validated 136-item food-frequency questionnaire was used to calculate the DII. The DII is based on scientific evidence about the relationship between diet and inflammatory biomarkers (C-reactive protein, IL-1β, IL-4, IL-6, IL-10 and TNF-α). Cox proportional hazard models were used to estimate hazard ratios (HR) and 95% confidence intervals (CI) for the association between the DII and incident cardiovascular disease (myocardial infarction, stroke or cardiovascular death).

**Results:**

The risk for cardiovascular events progressively increased with each increasing quartile of DII (p_trend_ = 0.017). The multivariable-adjusted HR for participants in the highest (most pro-inflammatory) vs. the lowest quartile of the DII was 2.03 (95% CI 1.06–3.88).

**Conclusions:**

A pro-inflammatory diet was associated with a significantly higher risk for developing cardiovascular events.

## Introduction

Cardiovascular disease (CVD) is considered the single most important cause of death worldwide and the second most common cause of death in high-income regions. In 2010, it was estimated that CVD caused 16 million deaths (30% of all death) and led to 293 million disability-adjusted life-years (DALYs) lost [1]. These data underline the need to identify factors that can be modified as part of effective preventive strategies, especially healthy lifestyle recommendations, to reduce cardiovascular risk.

During the last decade, evidence based on basic and clinical investigations has demonstrated a fundamental role for inflammation in atherogenesis [2]. Inflammatory factors identified contribute to the atherothrombotic process and the plaque rupture that underlies many acute vascular events. Research combining inflammatory biomarkers with vascular imaging support the systemic and diffuse nature of inflammation associated with cardiovascular events [3,4]. Moreover, multiple studies have shown that low-grade systemic inflammation leads to an increased risk of cardiovascular disease [4].

A large body of evidence has shown associations between diet and regulation of inflammation (C-reactive protein and other pro-inflammatory cytokines) leading to a probable modulation of the atherogenesis process and endothelial function [5–8]. Protective cardiovascular interventions such as increasing physical activity, avoiding smoking and sedentary behaviours or promoting healthy dietary patterns, could be mediated, at least partly, through anti-inflammatory effects [9].

In light of the important role of inflammation in atherogenesis, and its potential modulation by diet, classifying individuals’ diets according to their inflammatory properties could yield important information about the links between diet, inflammation, and CVD [10,11]. The dietary inflammatory index (DII) is a new, validated tool to quantify the inflammatory potential of a diet.

Our aim was to prospectively assess the association between the DII and the risk of CVD in a large cohort of Mediterranean middle-aged adults.

## Methods

### Study population

The SUN [Seguimiento Universidad de Navarra (University of Navarra follow-up)] study is an ongoing, multipurpose, prospective and dynamic cohort of university graduates conducted in Spain to assess associations between diet and lifestyles and the incidence of several diseases and mortality. The study design, methods and the cohort profile have been published in detail elsewhere [12]. Briefly, beginning in December 1999, highly educated participants, all of them university graduates, were contacted biennially. Enrolment is permanently open and follow-up is conducted through mailed questionnaires about lifestyle factors and medical conditions. Non-respondents are sent up to 5 additional mailings requesting their follow-up questionnaire.

**Fig 1:**
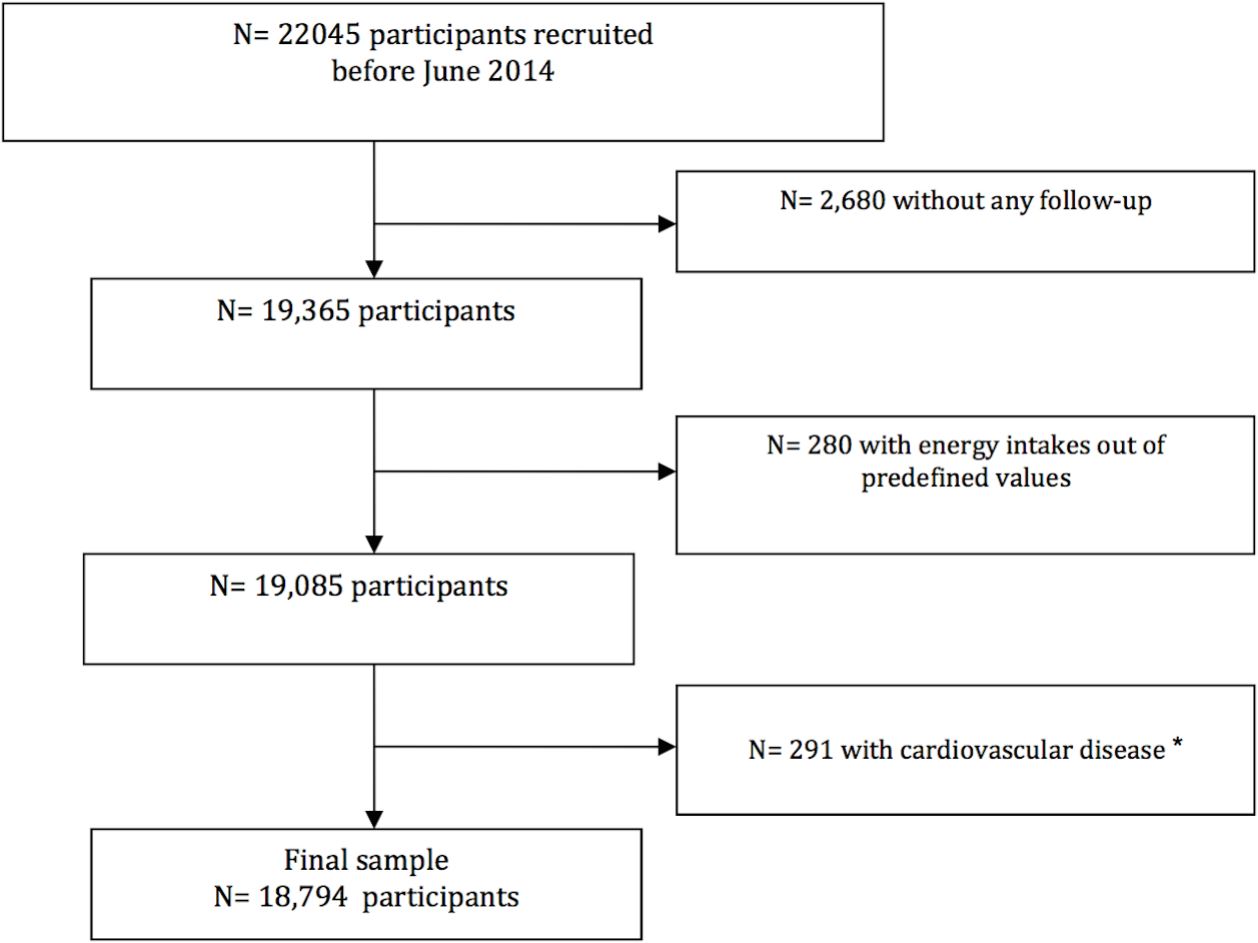
Flow chart of participants. **The SUN cohort.** * Cardiovascular disease: stroke, angina, myocardial infarction, and coronary artery revascularization.

Through June 2014, 22,045 participants had been recruited and had completed the baseline questionnaire of the SUN project. Among them, 19,365 participants had follow-up information (at least one follow-up questionnaire), achieving a retention rate of 87.8% Participants who did not meet our predefined limits for total energy intake (men: <400 kcal/day or >6,000; women: <400 or >5000 kcal/day (n = 280) and those who reported at baseline either coronary heart disease or stroke (n = 291) were excluded from the present analyses. Thus, the effective sample size was 18,794 participants (Fig 1).

The Institutional Review Board of the University of Navarra approved the study protocol. Voluntary completion of the baseline questionnaire was considered to imply informed consent.

### Dietary assessment

Dietary habits at baseline were assed using a 136-item, semi-quantitative food-frequency questionnaire (FFQ) previously validated in Spain [13]. Nutrient scores were calculated based on the frequency of intake of specified portion sizes for each food item. A trained dietician updated the nutrient data bank using the latest available information included in food composition tables for Spain [14,15].

Adherence to the Mediterranean diet was appraised according to the score proposed by Trichopoulou et al [16].

### The Dietary inflammatory index (DII)

The design and development of the DII has been described elsewhere [17]. Briefly, the DII is based on an extensive review of the literature published from 1950 to 2010 linking 1943 articles to a total of forty-five food parameters including various macronutrients, micronutrients, flavonoids and individual food items. The inflammatory potential for each food parameter was scored according to whether it increased (+1), decreased (-1) or had no effect (0) on six inflammatory biomarkers (IL-1β, IL-4, IL-6, IL-10, TNF-α and C-reactive protein). A z-score for each food consumed was calculated by subtracting the “standard global mean” from the amount reported and dividing this value by the standard deviation. To minimize the effect of “right skewing”, this value was then converted to a centered percentile score. The centered percentile score for each food parameter for each participant was then multiplied by the respective food parameter effect score, which was derived from the literature review, in order to obtain a food parameter-specific DII score for a given participant. All of the food parameter-specific DII scores were then summed to create the overall DII score for each participant in the study. The greater the DII score, the more pro-inflammatory the diet. More negative values represent more anti-inflammatory diets. The DII score could range from-8.87 (maximally anti-inflammatory) to +7.98 (maximally pro-inflammatory).

Construct validation of the DII was performed using data derived from two different sources of dietary intake information and serum high-sensitivity C-reactive protein (CRP) as the construct validator [17]. Thus far, the DII has been found to be associated with inflammatory cytokines including CRP and IL-6 [18], anthropometric measures of obesity [19], and various inflammation-related diseases [20–23].

In this study, a total of 28 food parameters considered in the DII score were derived from the FFQ and therefore could be used to calculate the DII. These include energy, carbohydrate, protein, total fat, alcohol, fiber, cholesterol, saturated fat, mono-unsaturated fat, poly-unsaturated fat, omega-3 fatty acids, omega-6 fatty acids, trans-fat, niacin, thiamin, riboflavin, vitamin B12, vitamin B6, iron, magnesium, selenium, zinc, vitamin A, vitamin C, vitamin D, vitamin E, folic acid and caffeine). The scoring for each food parameters used to calculate the DII is shown in S1 Table.

### Assessment of covariates

The baseline questionnaire requested information about lifestyle, medical history, anthropometric characteristics, sociodemographic factors and clinical variables. Physical activity was assessed with a previously validated questionnaire [24]. Metabolic equivalents (METs) were estimated to yield METs-h/week scores for each participant. Accuracy of self-reported weight and height for body mass index (BMI) calculation has been validated previously in a subsample of this cohort [25].

### Ascertainment of incident cardiovascular events

The primary end point for the present analysis was the composite of cardiovascular death, incident non-fatal acute coronary syndromes (myocardial infarction with or without ST elevation) or incident non-fatal stroke. Participants who reported any of these diagnoses on a follow-up questionnaire were asked for their medical records. An expert panel of physicians adjudicated the events by reviewing the medical records. The “third universal definition for myocardial infarction” was applied for non-fatal coronary syndromes [26]. Non-fatal stroke was defined as a focal-neurological deficit of sudden onset and via vascular mechanism that lasted more than 24 h. Deaths were reported to our research team by the subjects’ next of kin, work associates and postal authorities. For participants lost to follow-up, the National Death Index was checked to identify deceased cohort members and to obtain cause of death. Cardiovascular deaths were confirmed, according to the International Classification of Diseases 10^th^ edition by a review of medical records with the permission of the next of kin.

### Statistical analysis

For descriptive purposes, we summarized continuous variables with means and standard deviations (SD) and categorical variables using percentages across quartiles of DII. We adjusted baseline variables for sex and age. Person-years of follow-up were calculated for each participant from the date of completion of the baseline questionnaire to the date of cardiovascular event, the date of death, or the date of return of the last follow-up questionnaire, whichever occurred first.

Hazard ratios (HR) and 95% confidence intervals (CI) were calculated using Cox proportional hazard models for survival analyses considering the lowest quartile of DII (most anti-inflammatory) as the reference category. In order to control for potential confounding factors, successive degrees of adjustment were use: 1) adjusted for sex, using age as underlying time variable and calendar year of entering the cohort as stratification variable; 2) additionally adjusted for baseline hypertension, dyslipidaemia, diabetes, smoking status (current smoker, former smoker, never smoked), family history of cardiovascular disease, total energy intake (quartiles), physical activity (quartiles), BMI (quartiles), educational level (4 categories) and previous history of other cardiovascular diseases (tachycardia, atrial fibrillation, aortic aneurysm, pulmonary embolism, deep vein thrombosis, peripheral artery disease, heart valve disease, implantation of pacemaker); and 3) additionally adjusted for the habit of between-meal snacking, following a special diet, hours spent sitting down (quartiles) and hours spent watching television (quartiles).

We conducted tests of linear trend (likelihood ratio test) assigning to each category of the DII the median of the respective quartile and used the resulting variable in models equivalent to those just described.

We used multivariable logistic regression models to assess the relationship between successive quartiles of DII and 2-year incidence of medically diagnosed hypertension and hypercholesterolemia. To avoid the likely bias by reverse causation and to obtain evidence on early development of atherosclerosis, we excluded all participants with a previous self-reported diagnosis at baseline of any CVD, diabetes, hypertension, hypercholesterolemia and also those who followed special diets or who gained >5kg of body weight in the 5 years before the baseline assessment. This two latter exclusions were applied to avoid the possibility that the baseline diet (and consequently the baseline DII score) may be displaced to a more anti-inflammatory diet as a consequence of the prescription of special diets because of a medical diagnosis of disease or as a consequence of a personal decision of the participant after he/she observed that he/she was gaining weight.

We used Nelson-Aalen curves to describe the incidence of cardiovascular disease during follow-up across quartiles of the DII. We adjusted survival curves for sex, age, hypertension, dyslipidaemia, diabetes, smoking status (3 categories), family history of cardiovascular disease and total energy intake using the inverse probability weighting method.

In order to test whether the known association between Mediterranean diet and cardiovascular disease [27,28] was mediated by the anti-inflammatory effects of the Mediterranean diet we calculated residuals of DII on the Trichopoulou score of adherence to the Mediterranean dietary pattern and calculated HR and 95% CI for cardiovascular events using the lowest quartile of these residuals of DII (most anti-inflammatory) as the reference category. To assess the degree of overlapping (inverse association) between the Trichopoulou score and the DII we calculated the Pearson’s correlation coefficient (and 95% CI) between these two dietary indexes.

Subgroups analyses and tests for interactions were conducted according to sex and BMI (cut-off = 25kg/m^2^). As sensitivity analyses, we estimated adjusted HRs under several assumptions: 1) excluding events that occurred within a short follow-up period (<1 months and <3 months); 2) including only cardiovascular events occurring during the first 5 years of follow up (to avoid the unrealistic assumption of long-term stability of diets); 3) adopting different limits for total energy intake; 4) excluding participants habitually consuming aspirin; and 5) excluding participants with some other cardiovascular disease at baseline.

All p values were two tailed, and significance was set at p<0.05. The statistical analyses were performed with STATA version12 SE (StataCorp, College Station, TX).

## Results

Table 1 shows the main characteristics of the study participants according to quartiles of the DII score. The median DII score was-1.86, ranging from a maximum anti-inflammatory value of-5.14 to a maximum pro-inflammatory value of +3.97. The mean (SD) age of the participants at recruitment was 38 (12) with 61% women. Only 4.5% of participants were > 60 years old. Only small differences between categories of DII in family history of cardiovascular disease, BMI, dyslipidaemia and marital status were observed. There were higher proportions of males and current smokers in the highest DII score quartiles (most pro-inflammatory dietary pattern). The reported physical activity, total energy intake, alcohol intake and prevalence of diabetes tended to decrease with increasing categories of DII.

**Table 1:** Age and sex adjusted baseline characteristics by quartiles of dietary inflammatory index score (DII) in the SUN study.

	Quartiles of dietary inflammatory index score			
	Q1	Q2	Q3	Q4
	Most anti-inflammatory			Most pro-inflammatory
Median DII	-3.18	-2.27	-1.40	0.30
(min, max)	(-5.14, -2.68)	(-2.68, -1.86)	(-1.86, -0.74)	(-0.74, 3.97)
n	4699	4698	4699	4698
Age (years)	39.6 (12.5)	38.8 (12.2)	37.6 (11.6)	37.1 (11.5)
Sex (% men)	37.2	35.1	37.5	47.1
Family history of CHD^*^, %	14	13.7	14	13.7
Hypertension^*^, %	7.4	7.2	6.3	6.8
Dyslipidaemia^*^, %	23.4	22.9	22.9	23.4
Diabetes^*^, %	1.9	1.9	1.6	1.2
Smoking^*^, %				
Never	48.6	48.3	46	43.4
Former	29.2	28.4	29.8	28.5
Current	19.6	20.7	21.5	25.6
Body mass index^*^, kg/m^2^	23.4	23.5	23.5	23.5
Physical activity, METs/week	26.3	22.7	20.6	17.2
Marital status^*^, %				
Single	46.9	43.8	41.9	43.4
Married	47.5	50.7	53.1	51.1
Other	4.8	4.8	4.3	4.7
Total energy intake^*^, kcal/d	3070	2630	2340	1894
Alcohol intake^*^, g/d	8.3	6.6	6.3	5.9
Total fat intake^*^, % energy	36.1	35.5	36.4	38.4
Saturated^*^, % energy	11.4	11.9	12.8	13.8
Monounsaturated^*^, % energy	15.5	15.2	15.7	16.5
Polyunsaturated^*^, % energy	5.7	5.1	4.9	5.1

SD: Standard Deviation; Q: Quartile; CHD: Coronary heart disease; METs: Metabolic equivalents.

*Adjusted by age and sex

### DII and risk of cvd

**Fig 2:**
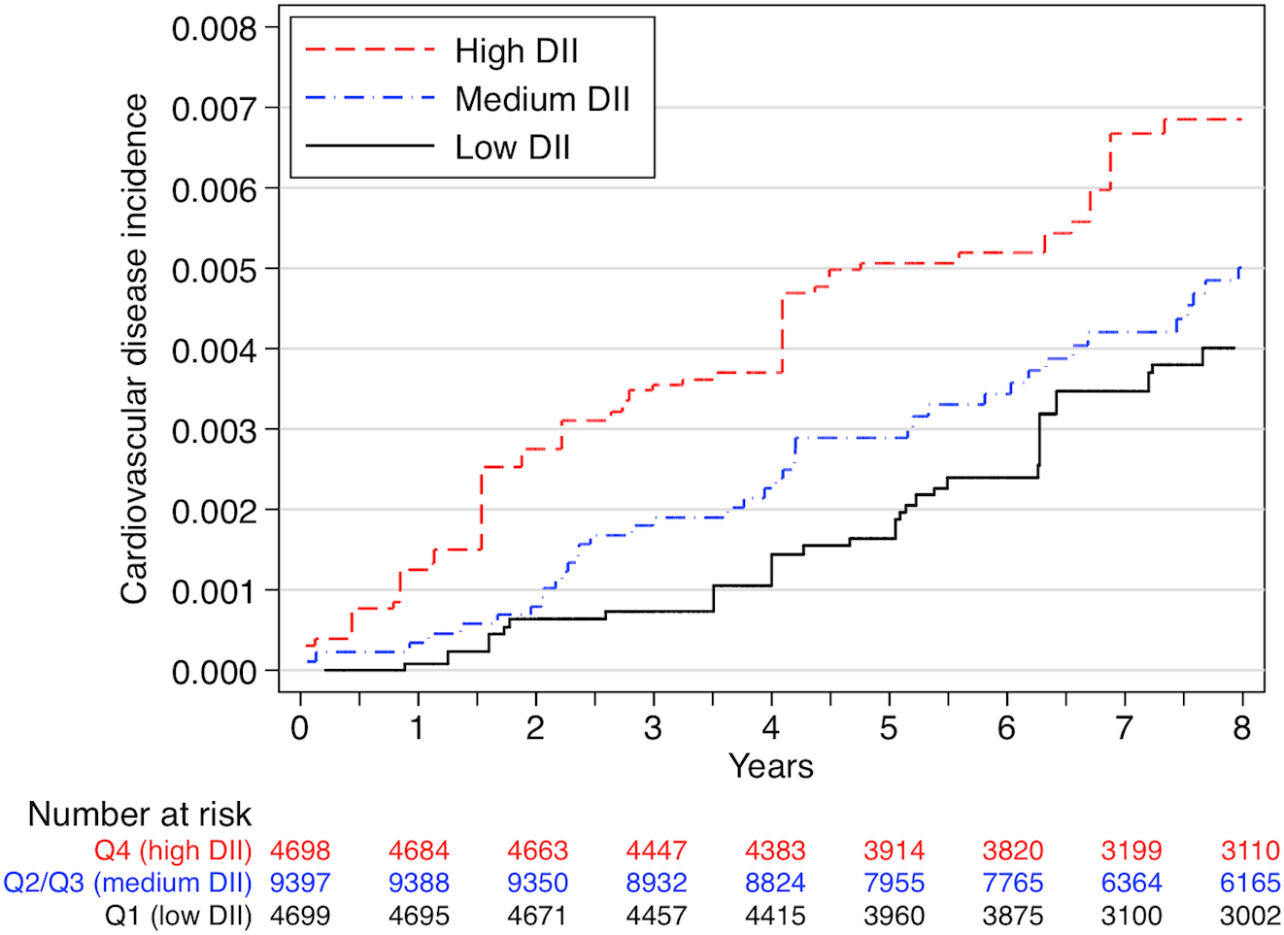
Nelson-Aalen estimates of incidence of CVD across quartiles of the DII. Adjusted for sex, age, hypertension, dyslipidaemia, diabetes, smoking status (3 categories), familiar history of cardiovascular disease and total energy intake, using inverse probability weighting. The two intermediate quartiles were merged to build the “medium” category.

During a median of 8.9 years of follow-up and 168,112 person-years at risk, 117 incident cases of CVD (41 non-fatal myocardial infarctions, 27 non-fatal strokes and 49 cardiovascular deaths) were registered. The incidence rate was 0.69 per 1000 person-years, ranging from 0.58 per 1000 person-years in the most anti-inflammatory quartile of DII to 0.87 in the most pro-inflammatory quartile of DII. Fig 2 shows the adjusted Nelson-Aalen curves for the incidence of CVD across quartiles of the DII.

We observed a direct association between DII and CVD. A higher, i.e., more pro-inflammatory, DII score was significantly associated with a higher CVD risk after adjustment for age and sex (Table 2). This direct association remained significant after adjustment for other potential risk factors. Comparing the highest vs. the lowest quartile of DII, the HR was 2.03 (95% CI 1.06–3.88; P-trend = 0.017) in the fully adjusted model.

**Table 2:** Hazard ratios (95% CI) for the risk of cardiovascular events, according to the dietary inflammatory index score, the SUN.

	Quartiles of dietary inflammatory index score				
	Q1	Q2	Q3	Q4	P for trend
	Most anti-inflammatory			Most pro-inflammatory	
Cases	24	24	32	37	
Person-years of follow up	41,240	42,496	42,029	42,345	
Incidence rate /1,000 person years	0.58	0.56	0.76	0.87	
(95% CI)	(0.39–0.87)	(0.38–0.84)	(0.54–1.08)	(0.63–1.2)	
Crude HR*	1 (ref)	1	1.62	1.98	0.003
		(0.57–1.79)	(0.94–2.8)	(1.16–3.36)	
HR adjusted for sex *	1 (ref)	1.02	1.52	1.75	0.016
		(0.57–1.81)	(0.88–2.63)	(1.03–2.98)	
Multivariable adjusted HR †	1 (ref)	1.05	1.75	1.95	0.018
		(0.58–1.89)	(0.96–3.18)	(1.02–3.72)	
Multivariable adjusted HR ‡	1 (ref)	1.05	1.75	2.03	0.017
		(0.58–1.9)	(0.96–3.19)	(1.06–3.88)	

*age as underlying time variable

† Additionally adjusted for cardiovascular risk factors (hypertension, dyslipidaemia, diabetes, smoking status (3 categories), family history of cardiovascular disease), total energy intake (quartiles), physical activity (quartiles), body mass index (quartiles) educational level (4 categories) and other cardiovascular diseases (tachycardia, atrial fibrillation, aortic aneurysm, pulmonary embolism, deep vein thrombosis, peripheral artery disease, heart valve disease, or pacemaker placement)

‡ Additionally adjusted for special diet at baseline, snacking, average time sitting (quartiles), average time spent watching television (quartiles).

We conducted several sensitivity analyses to assess the robustness of our results under various scenarios (Table 3). When we excluded cardiovascular events diagnosed during the first month of follow up, we found a similar association: the HR comparing extreme quartiles of DII was 1.98 (95% CI, 1.03–3.8). We observed a slightly attenuated association when we excluded CVD cases that occurred within the fist 3 months of follow up: HR 1.89 (95% CI, 0.98–3.64). When we repeated the analyses including only cardiovascular events diagnosed during the first 5 years of follow up, the HR (for the comparison between the highest and lowest quartile) was 3.45 (95% CI, 1.36–8.79). Results showed that the associations were similar when we set tighter limits for total energy intake (men: 800–4000 kcal/day, women: 500–3500 kcal/day) [29] (HR = 2.10; 95%CI, 1.09–3.67). When we excluded participants with some other cardiovascular disease at baseline (tachycardia, atrial fibrillation, aortic aneurysm, pulmonary embolism, deep vein thrombosis, peripheral artery disease, heart valve disease, or pacemaker placement) the HR was 1.99 (95% CI, 0.98–4) and when we excluded participants with hypertension the HR was 2.33 (95% CI, 1.07–5.07). All of these analyses showed a significant dose-response relationship (p for trend < 0.05). The association was attenuated and became non-significant after excluding participants with chronic aspirin intake (HR = 1.88; 95% CI, 0.96–3.67) or participants with chronic non-aspirin analgesics intake (HR = 1.68; 95% CI, 0.84–3.34).

**Table 3:** Sensitivity analysis Hazard ratios (95% CI) ^*^ for the risk of cardiovascular events between extreme quartiles of dietary inflammatory index score (the SUN).

	cases	n	Q4 vs. Q1 (ref.)	p for trend †
Excluding cardiovascular events during the first month of follow up	115	19,782	1.98 (1.03–3.8)	0.022
Excluding cardiovascular events during the first 3 months of follow up	113	18,790	1.89 (0.98–3.64)	0.033
Including only cardiovascular events during the first 5 years of follow up	57	18,794	3.45 (1.36–8.79)	0.004
Setting tighter energy limits	118	19,069	2.10 (1.09–4.03)	0.011
Excluding participants with other cardiovascular disease	96	18,184	1.99 (0.98–4.0)	0.021
Excluding participants with hypertension at baseline	77	17,273	2.33 (1.07–5.07)	0.007
Excluding participants with chronic aspirin intake	109	18,110	1.88 (0.96–3.67)	0.026
Excluding patients with asthma at baseline	81	15,705	2.60 (1.16–5.82)	0.010
Excluding participants with non-aspirin analgesics intake	104	16,488	1.68 (0.84–3.34)	0.064

* Age as underlying time variable. Adjusted for sex, cardiovascular risk factors (hypertension, dyslipidaemia, diabetes, smoking status (3 categories), family history of cardiovascular disease), total energy intake (quartiles), physical activity (quartiles), body mass index (quartiles) educational level (4 categories), other cardiovascular diseases, special diet at baseline, snacking, average time sitting (quartiles), average time spent watching television (quartiles).

† For the 4 quartiles

When we stratified by sex, the HR for CVD among men comparing extreme quartiles of the DII score was 2.40 (95% CI, 1.14–5.04; P for trend = 0.01). However, this association was not evident for women (HR = 0.79; 95% CI, 0.17–3.72; P for trend = 0.99). This lack of association can be probably attributed to the small number of incident cases among women (22 cases). This low incidence of CVD in women was to be expected because in our cohort, women were younger than men (mean age for women at baseline was 35.4 years vs. 42.6 years for men) and women tend to have low rates of CVD at younger ages. In any case, we did not observe any evidence of a modification of effect of the DII on CVD by sex (P for interaction 0.28). Among participants who were overweight or obese (BMI > 25kg/m^2^) the HR was 2.79 (95%CI 1.14–6.86) whereas among those without overweight or obesity the HR was 1.29 (95%CI 0.47–3.56) (P for interaction = 0.47).

### DII and risk of hypertension and hypercholesterolemia

In order to explore the association between DII and other intermediate predictors of cardiovascular disease, we estimated multivariable-adjusted OR for the risk of hypertension or hypercholesterolemia after 2-year follow-up across quartiles of the DII (Table 4). A statistically significant association was found between DII and hypertension; however, no association was apparent for hypercholesterolemia.

**Table 4:** Odds ratio (95% CI) for the incidence of hypertension and hypercholesterolemia at 2-year follow up, according to baseline dietary inflammatory index score. The SUN project.^1^

	Quartiles of dietary inflammatory index score				
	Q1	Q2	Q3	Q4	P for trend
	Most anti-inflammatory			Most pro-inflammatory	
**Hypertension**					
Sex and age adjusted OR	1 (ref)	1.19	1.61	1.47	0.026
		(0.82–1.73)	(1.13–2.30)	(1.02–2.11)	
Multivariable adjusted OR^2^	1 (ref)	1.25	1.76	1.67	0.018
		(0.85–1.84)	(1.19–2.59)	(1.09–2.57)	
Multivariable adjusted OR^3^	1 (ref)	1.26	1.78	1.71	0.013
		(0.85–1.85)	(1.20–2.62)	(1.11–2.64)	
**Hypercholesterolemia**					
Sex and age adjusted OR	1 (ref)	1.09	0.83	1.19	0.42
		(0.77–1.53)	(0.57–1.20)	(0.85–1.68)	
Multivariable adjusted OR^2^	1(ref)	1.02	0.74	1.03	0.98
		(0.72–1.46)	(0.50–1.10)	(0.69–1.55)	
Multivariable adjusted OR^3^	1(ref)	1.03	0.74	1.04	0.95
		(0.72–1.47)	(0.50–1.10)	(0.69–1.57)	

1 Excluding subjects with diagnosis at baseline of diabetes, hypertension, hypercholesterolemia, use of special diets or other cardiovascular diseases (tachycardia, atrial fibrillation, aortic aneurysm, pulmonary embolism, deep vein thrombosis, peripheral artery disease, heart valve disease, or pacemaker placement). Subjects with weight gain > 5kg in the previous 5 years were also excluded

2 Additionally adjusted for: family history of cardiovascular disease, smoking status, total energy intake (quartiles), physical activity (quartiles), body mass index (quartiles)

3 Additionally adjusted for: educational level (4 categories), total alcohol intake (quartiles), snaking, average time sitting (quartiles), average time spent watching television (quartiles)

We also conducted stratified analyses to assess whether the association between DII and hypertension varied by sex. The fully-adjusted multivariable OR across quartiles of DII was 2.93 for women (95% CI 1.48–5.81) (p for trend = 0.001) and 1.20 (95% CI 0.68–2.11) (p for trend = 0.460) for men.

### Association between DII and Mediterranean diet

Finally, in order to determine whether the known inverse association of the Mediterranean diet with cardiovascular disease was mediated by the DII, we computed residuals by regressing the DII on the Mediterranean diet score (Trichopoulou). The multivariable-adjusted HR across quartiles of residuals of DII was 0.77 (95% CI 0.43–1.39), 0.88 (95% CI 0.48–1.60), and 1.10 (95% CI 0.60–2.01) (p for trend 0.45). A moderate inverse association between the Trichopoulou score and the DII was apparent with Pearson’s r = -0.59 (95% CI-0.60 to-0.58).

## Discussion

In this study based on the prospective SUN cohort, we found that higher values of the DII (representing the most pro-inflammatory dietary potential) were associated with a significantly increased risk of CVD independent of other lifestyle CVD risk factors. Furthermore, we found consistent robust results in various sensitivity analyses. This apparent direct association was stronger when we included cardiovascular events diagnosed during only the first 5 years of follow up. It was also stronger in men and among overweight/obese participants. Finally, a more pro-inflammatory DII (i.e., higher) was associated with incident hypertension but not with hypercholesterolemia.

Prior research has shown that inflammation plays an important role in cardiovascular disease; indeed, atherosclerosis is considered to be an inflammatory process. Different inflammatory markers such as C-reactive protein (CRP) or pro-inflammatory cytokines such as interleukin-6 or interleukin-1 have been shown to be elevated in patients with myocardial infarction or unstable angina, reflecting low-grade inflammatory status in the vascular bed [30,31]. A recently published study using intracoronary imaging in patients who underwent coronary angiography demonstrated that high CRP concentrations were associated with a higher coronary plaque burden and the presence of large lesions. They also were predictive of a higher rate of cardiovascular events [4]. Moreover, only moderate elevation of CRP on a highly sensitive immunoassay has been postulated to be an independent risk factor in coronary artery disease in healthy populations, similar to blood pressure or elevated cholesterol [32,33]. To the best of our knowledge, at least two large placebo-controlled trials using targeted anti-inflammatory agents to reduce cardiovascular events have been initiated to confirm this hypothesis [34–36] reflecting the increasing interest in the relation between inflammation and cardiovascular disease.

We recently showed the association between the DII and the incidence of CVD in a very different population composed of elderly participants at high cardiovascular risk and with a low educational level from the PREDIMED study [37]. The association between diet and inflammatory markers has been evaluated in previous research [5–11, 38–47]. Several studies have shown that meat-based or “Western” dietary patterns tend to be positively associated with inflammatory biomarkers, while vegetable or fruit consumption are inversely associated [38–40]. Specifically, the Mediterranean diet has been associated with improvements in inflammatory biomarkers, especially in CRP [27, 46–50]. The pathways through which a healthy dietary pattern can reduce low-grade inflammatory status are unclear. The effect of dietary fiber intake on cytokine production, the anti-inflammatory effects of some fatty acids, and the improved endothelial function related to antioxidant vitamins have been postulated as potential mechanisms [51].

Our observed direct association disappeared when we used as exposure the residuals of the DII on the Mediterranean dietary score. These residuals can be interpreted as the variability in the dietary pattern captured by the DII that is not explained by the Mediterranean diet score. These DII residuals are no longer correlated with the Mediterranean diet. The null value of the HR of this model can be interpreted as the estimated cardiovascular effect of the residual inflammatory properties of diet that are not explained by adherence to a Mediterranean diet. The lack of significance for this residual of the DII supports the idea that the important issue is the inverse correlation between the DII and the Mediterranean diet scores. They were computed in different ways because the input for the DII were macro and micronutrients whereas the input for the Mediterranean score were mainly food groups. Both scores were inversely correlated with a moderate strength. The fact that the residual effect was not significant suggest that inflammation is likely to be the major pathway through which diet exerts its effect in this study. The anti-inflammatory properties of the Mediterranean diet can explain the successful results of the large PREDIMED randomized trial [48] in consistency with many large observational studies. In fact, when we assessed the effect of the Mediterranean dietary score on cardiovascular events, the adjustment for the DII attenuated the inverse association [the HR for the Trichopoulou score of adherence to Mediterranean diet was 0.68 (95% CI 0.54–0.86), and after adjustment for DII (quartiles) the HR was 0.73 (95% CI 0.55–0.96)].

The usefulness of the DII is to focus on the effect that diet may have according to its inflammatory properties. In fact, the DII has been associated with known inflammation-related conditions such as obesity [19], asthma and FEV_1_ in an Australian population [20], colorectal cancer among women in the Iowa Women’s Health Study [21], the Women’s Health Initiative [52] and in a Spanish case-control study [53], and prostate cancer [22], and pancreatic cancer [23] in an Italian case-control study.

A limitation in our study is its reliance on dietary self-report. Food-frequency questionnaires (FFQ) are known to contain a certain degree of measurement error, which might affect results that depend on such evaluation. Reliability and validity of the FFQ used in our cohort has been extensively evaluated, showing good correlation with nutrient intake according to repeated food records [13]. Therefore, we do not think that misclassification might be an alternative explanation for the significant association we identified. Second, we acknowledge that the small number of observed cardiovascular events may be another limitation of our study. In our cohort participants were young, predominantly women, with high educational level and low prevalence of obesity and other cardiovascular risk factors. Despite this limitation which might reduce statistical power, we found significant associations between the inflammatory potential of diet and cardiovascular events. Our results reinforce earlier findings reported in a cohort of elderly subjects at high cardiovascular risk and with a lower educational level [37]. Now by including a young population and finding similar results, our study contributes to suggest that the DII is causally related to CVD both among middle-age and elderly adults.

Some relevant characteristics of our study deserve to be highlighted. These include: a large sample size, a cohort design with a long-term follow-up, inclusion of multiple variables as potential confounders, use of validated questionnaires, medical confirmation of cardiovascular events, and existence of published validation studies of our assessment methods.

## Conclusions

Our results provide evidence supporting that a higher DII score (indicating a more pro-inflammatory diet) is directly associated with cardiovascular events. These results suggest the importance of promoting dietary patterns with low inflammatory potential for the general population. Further randomized interventional studies analysing high-risk population for cardiovascular events are warranted to confirm the cardiovascular protection exerted by diets with a low inflammatory potential.

## Supporting Information

S1 Table Scoring for each food parameters used to calculate the DII. (DOCX)
