# Supplementary material for: Dietary Inflammatory Index and Incidence of Cardiovascular Disease in the SUN Cohort
Source: PLoS One. 2015 Sep 4;10(9):e0135221. doi: 10.1371/journal.pone.0135221 (PMC4560420; doi:10.1371/journal.pone.0135221)
Supplement: S1 Table — (DOCX) [file pone.0135221.s001.docx]

S1 Table 1. Scoring for each food parameters used to calculate the DII

| **Food Parameter** | **Overall inflammatory effect score** |
| --- | --- |
| Energy (kcal) | 0.180 |
| Carbohydrate (g) | 0.097 |
| Total fat (g) | 0.298 |
| Alcohol (g) | -0.278 |
| Fiber (g) | -0.663 |
| Protein (g) | 0.021 |
| Vitamin B12 (μg) | 0.106 |
| Vitamin B6 (mg) | -0.365 |
| Beta Carotene (μg) | -0.584 |
| Omega 3 (g) | -0.436 |
| Omega 6 (g) | -0.159 |
| Mono-unsaturated fatty acid (g) | -0.009 |
| Saturated fatty acid (g) | 0.373 |
| Trans fat (g) | 0.229 |
| Iron (mg) | 0.032 |
| Polyunsaturated fatty acid (g) | -0.337 |
| Riboflavin (mg) | -0.068 |
| Thiamin (mg) | -0.098 |
| Niacin (mg) | -0.246 |
| Vitamin A (RE) | -0.401 |
| Magnesium (mg) | -0.484 |
| Zinc (mg) | -0.313 |
| Selenium (μg) | -0.191 |
| Vitamin C (mg) | -0.424 |
| Vitamin D (μg) | -0.446 |
| Vitamin E (mg) | -0.419 |
| Folate (μg) | -0.190 |
| Caffeine (g) | -0.110 |
